# Funding community collaboration to develop effective therapies for neurofibromatosis type 1 tumors

**DOI:** 10.15252/emmm.201911656

**Published:** 2019-12-02

**Authors:** Salvatore La Rosa, Vidya Browder, Annette C Bakker, Jaishri O Blakeley, Sharad K Verma, Ling M Wong, Jill Morris, Naba Bora

**Affiliations:** ^1^ Children's Tumor Foundation New York NY USA; ^2^ Johns Hopkins University Baltimore MD USA; ^3^ National Institute of Neurological Disorders and Stroke Bethesda MD USA; ^4^ Congressionally Directed Medical Research Programs Fort Detrick MD USA

**Keywords:** Cancer

## Abstract

The time from identifying a drug target to a new drug approval is often measured in decades and can take even longer for therapies to treat rare diseases. In fact, 95% of rare diseases do not have a specific therapy approved at all. Coordinated efforts to augment the drug development pipeline along with long‐term and comprehensive support that enable scientific breakthroughs for rare diseases are possible, but it requires integration across multiple stakeholders. This article analyzes the coordinated funding efforts of four federal and philanthropic organizations to advance drug development for neurofibromatosis type 1‐associated tumors and discusses how these organizations have been collaborating and evolved practices to optimize funding and research support.

## Graphical abstract

La Rosa *et al* analysed coordinated funding efforts of four federal and philanthropic organizations to advance drug development for neurofibromatosis type 1‐associated tumors and discuss how these organizations have been collaborating and evolved practices to optimize funding and research support.

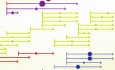

Neurofibromatosis type 1 (NF1) is an autosomal dominant genetic syndrome caused by mutations in the *NF1* gene (Gutmann *et al*, 2017). The hallmarks of NF1 are development of multiple tumors, including plexiform neurofibromas (pNF), cutaneous neurofibromas (cNF), and gliomas. The syndrome has multiple manifestations, and the clinical severity of NF1 is highly variable, with significant differences even within families sharing a mutation. NF1 patients are also at increased risk of developing additional malignancies such as peripheral nerve sheath tumors (MPNST) and breast cancer (Frayling *et al*, 2018). Despite the significant morbidity, therapeutic developments have been slow, and there are currently no approved therapies for the most common tumors associated with NF1. Surgical removal of tumors is the current “standard of care” for pNFs, but this is rarely feasible without significant morbidity or incomplete resection. In contrast, surgery is rarely performed for NF1 optic pathway gliomas given the risk of permanent vision loss. Symptomatic gliomas are typically managed with chemotherapeutic agents, which, though often effective, are associated with long‐term sequelae such as cognitive dysfunction (de Blank *et al*, 2016). Thus, an effective, long‐term therapy for NF1‐associated tumors is a major unmet medical need.

The *NF1* protein product, neurofibromin, negatively regulates Ras signal‐transduction pathways. Several preclinical studies validated mitogen‐activated protein kinase (MEK), a component of the Ras‐Raf‐MEK‐ERK signaling cascade, as a potential therapeutic target for NF1 tumors and other manifestations. At least four MEK inhibitors, mirdametinib (PD0325901), trametinib (GSK1120212, Mekinist), binimetinib (ARRY‐438162, MEK162), and selumetinib (AZD6244), have progressed to clinical trials in NF1 patients and, at the time of writing, ClinicalTrials.gov lists 23 trials to evaluate these MEK inhibitors for various manifestations of NF1 (Table 1). Selumetinib was granted Orphan Drug Designation and Breakthrough Therapy Designation by the US Food and Drug Administration (FDA) and the European Medicines Agency (EMA) based on encouraging results in pediatric NF1 patients with inoperable pNFs (Dombi *et al*, 2016). If approved, selumetinib will be the first drug to treat NF1‐associated tumors.

**Table 1:** Ongoing MEK inhibitor clinical trials for conditions including NF1^a^

ClinicalTrials.gov identifier	Intervention	Study name	Phase	Recruitment status
NCT02096471	PD‐0325901	MEK Inhibitor PD‐0325901 Trial in Adolescents and Adults With NF1 (MEK Inhibitor)	Phase 2	Completed
NCT03962543	Mirdametinib (PD‐0325901)	MEK Inhibitor Mirdametinib (PD‐0325901) in Patients With Neurofibromatosis Type 1‐Associated Plexiform Neurofibromas	Phase 2	Not Yet Recruiting
NCT02124772	Trametinib, Dabrafenib	Study to Investigate Safety, Pharmacokinetic (PK), Pharmacodynamic (PD) and Clinical Activity of Trametinib in Subjects With Cancer or Plexiform Neurofibromas and Trametinib in Combination With Dabrafenib in Subjects With Cancers Harboring V600 Mutations	Phase 1/2a	Recruiting
NCT03363217	Trametinib	Trametinib for Pediatric Neuro‐oncology Patients With Refractory Tumor and Activation of the MAPK/ERK Pathway	Phase 1/2	Recruiting
NCT03190915	Trametinib	Trametinib in Treating Patients With Relapsed or Refractory Juvenile Myelomonocytic Leukemia	Phase 2	Recruiting
NCT03232892	Trametinib	Trametinib in Patients With Advanced Neurofibromatosis Type 1 (NF1)‐Mutant Non‐Small‐Cell Lung Cancer	Phase 2	Recruiting
NCT02465060	Trametinib	Targeted Therapy Directed by Genetic Testing in Treating Patients With Advanced Refractory Solid Tumors, Lymphomas, or Multiple Myeloma (The MATCH Screening Trial)	Phase 2	Recruiting
NCT03741101	Trametinib	Treatment of NF1‐related Plexiform Neurofibroma With Trametinib	Phase 2	Recruiting
NCT01885195	Binimetinib	MEK162 for Patients With RAS/RAF/MEK‐Activated Tumors (SIGNATURE)	Phase 2	Completed
NCT03231306	Binimetinib	Phase II Study of Binimetinib in Children and Adults With NF1 Plexiform Neurofibromas (NF108‐BINI)	Phase 2	Recruiting
NCT02285439	Binimetinib	Phase I/II Study of MEK162 for Children With Ras/Raf Pathway‐Activated Tumors	Phase 1/2	Recruiting
NCT01089101	Selumetinib	Selumetinib in Treating Young Patients With Recurrent or Refractory Low‐Grade Glioma	Phase 1/2	Recruiting
NCT01362803	Selumetinib	AZD6244 Hydrogen Sulfate (Selumetinib) for Children With Nervous System Tumors	Phase 2	Active, not recruiting
NCT02407405	Selumetinib	Selumetinib in Treating Patients With Neurofibromatosis Type 1 and Plexiform Neurofibromas That Cannot Be Removed by Surgery	Phase 2	Recruiting
NCT02839720	Selumetinib	Selumetinib in Treating Patients With Neurofibromatosis Type 1 and Cutaneous Neurofibroma	Phase 2	Recruiting
NCT03109301	Selumetinib	Mitogen‐Activated Protein Kinase Kinase (MEEK/2) Inhibitor Selumetinib (AZD6244 Hydrogen Sulfate) in People With Neurofibromatosis Type 1 (NF1) Mutated Gastrointestinal Stromal Tumors (GIST)	Phase 2	Withdrawn
NCT03213691	Selumetinib	Selumetinib in Treating Patients With Relapsed or Refractory Advanced Solid Tumors, Non‐Hodgkin Lymphoma, or Histiocytic Disorders With Activating MAPK Pathway Mutations (A Pediatric MATCH Treatment Trial)	Phase 2	Recruiting
NCT03259633	Selumetinib	An Intermediate Access Protocol for Selumetinib for Treatment of Neurofibromatosis Type 1	Expanded access	Available
NCT03326388	Selumetinib	Intermittent Dosing Of Selumetinib In Childhood NF1‐Associated Tumors (INSPECT)	Phase 1/2	Not yet recruiting
NCT03649165	Selumetinib	A Study to Evaluate Bioavailability and Food Effect of Selumetinib (AZD6244) in Healthy Male Participants	Phase 1	Completed
NCT03433183	Selumetinib	SARC031: MEK Inhibitor Selumetinib (AZD6244) in Combination With the mTOR Inhibitor Sirolimus for Patients With Malignant Peripheral Nerve Sheath Tumors	Phase 2	Recruiting
NCT03871257	Selumetinib, Carboplatin/Vincristine	A Study of the Drugs Selumetinib versus Carboplatin/Vincristine in Patients With Neurofibromatosis and Low‐Grade Glioma	Phase 3	Not yet recruiting
NCT03975829	Trametinib Dabrafenib	Pediatric Long‐Term Follow‐up and Rollover Study	Phase 4	Not yet recruiting

a Search done on clinicaltrial.gov on August 26, 2019.

## Funding landscape in NF

The funding landscape of NF1‐MEK research, and of NF1 research as a whole, has been shaped by the efforts of multiple federal and philanthropic organizations worldwide. In addition to the National Institutes of Health (NIH, intramural and extramural), the organizations and research programs that have played a crucial role in advancing NF1‐MEK research include the Department of Defense (DoD), Congressionally Directed Medical Research Programs (CDMRP), Children's Tumor Foundation (CTF), and Neurofibromatosis Therapeutic Acceleration Program (NTAP) at Johns Hopkins University.

This report presents a retrospective analysis of the NF1‐MEK funding data for the years 2006–2017 including, but not limited to, funding from these organizations. Through this analysis, we illustrate how their contributions during the past 10 years have intentionally created workflows that leverage the efforts of each group to maximize resources and accelerate the pace of research.

The data were obtained from Dimensions for Funders (https://www.dimensions.ai/), a database of publicly and privately funded research projects worldwide, supplemented with iSearch, a portfolio analysis tool internal to NIH, and Research, Condition, and Disease Categorization (RCDC), a classification scheme used by the NIH (NIH, 1997; NIH, 2011). The search results were manually inspected for relevance to MEK inhibition to treat *NF1*, and the Broad Research Area (BRA) classification implemented in Dimensions was used to distribute the grants into two categories: “basic research” and “clinical research” (see Appendix Supplementary Methods; Table 2).

**Table 2:** Federal and philanthropic research organizations providing NF1‐MEK research funding in 2006–2017

Funder	Grants in 2006–2017		Basic research		Clinical research	
	Number	Funding (US$)	Number	Funding (US$)	Number	Funding (US$)
NIH	29	51,815,350	17	25,114,554	12	26,700,796
CDMRP	17	38,448,301	7	4,004,176	10	31,444,125
CTF	34	6,020,624	20	1,374,947	14	4,645,677
NTAP	5	3,755,487	–	–	5	3,755,487
CTF/NTAP (co‐funding)	1	3,658,649	–	–	1	3,658,649
Others	12	1,629,866	7	547,708	5	1,082,158
Total	98	102,328,277	51	31,041,385	47	71,286,892

**Figure 1:**
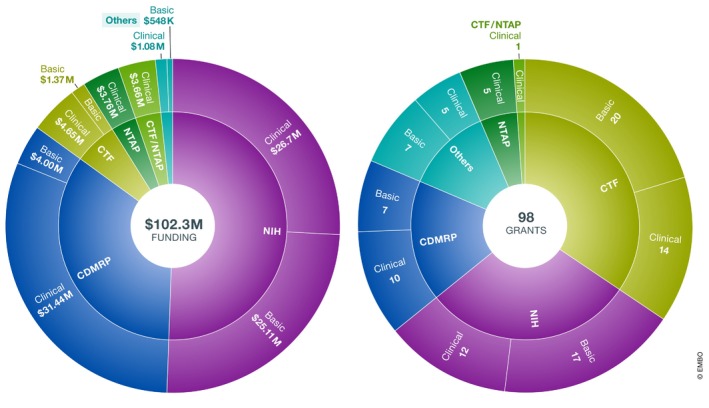
Total funding across organizations for NF1‐MEK research in 2006–2017 (A) Funding amounts. (B) Number of grants.

We identified a total of 98 relevant grants between 2006 and 2017 with an aggregate funding of US$102.33 million. Of these, 86 grants totaling US$100.7 million were funded by the NIH, CDMRP, CTF, and NTAP. The remaining 12 grants representing a total volume of US$1.6 million were awarded by the Japan Society for the Promotion of Science, Melanoma Research Alliance, St. Baldrick's Foundation, and National Natural Science Foundation of China (Fig 1).^1^

**Figure 2:**
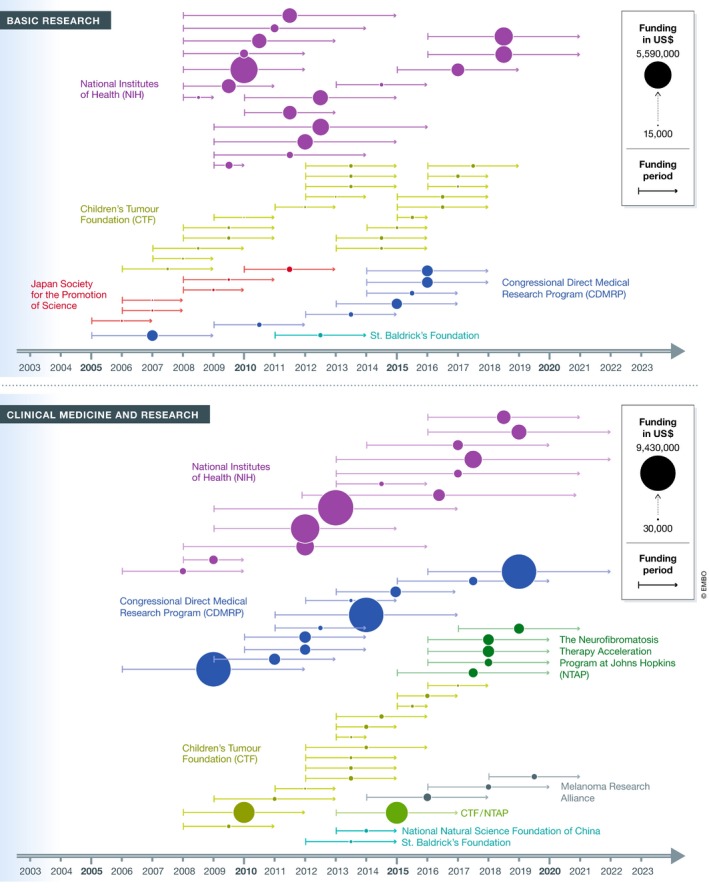
Timeline of NF1‐MEK research awards by funding organizations Bubble size is proportional to the grant size, and each grant is placed on the timeline based on its award period and grouped by organization. Almost all grants are multi‐year funding; funding period for each grant is displayed with a year start–year end line.

As shown in the detailed analysis (see Appendix Supplementary Methods), the major NF funders (NIH, CDMRP, CTF, and NTAP) have played distinct and complementary roles in developing a MEK inhibitor to treat NF1‐associated tumors. While NIH and CDMRP have provided the bulk of research dollars across all stages of research, CTF has “seed‐funded” NF research with a steady stream of small grants for basic and translational research as well as awards to attract investigators to the field (Fig 2). In fact, from a limited analysis conducted on all grants assigned by CTF in the years 2010–2014, it emerges that CTF's awardees were able to use the data generated by these awards to apply for NIH and CDMRP funding. Specifically, from an aggregate US$16 million awarded by CTF from 2010 to 2014, awardees obtained at least US$38 million follow‐up funding to continue their research. At the same time, partnerships for preclinical translational projects such as the NFPC (NF Preclinical Consortium) and NFTC (NF Therapeutic Consortium) allowed researchers to gather essential information and data to launch clinical studies (Maertens *et al*, 2017).

There are several important observations from this analysis: The importance of communication between funding agencies, the critical role of philanthropies, and the realization that integration of funding schemes are feasible and beneficial for research.

## Communication

In 2006, the major federal funding programs coordinated their funding commitment to focus their resources either on basic research (NIH) or on clinical trials (NF Research Program, NFRP/CDMRP). While both agencies have funded critical projects across the whole spectrum from basic discovery to clinical trial endpoints, NIH devoted their efforts to basic and translational research, while NFRP committed to fund the NF Clinical Trials Consortium so as to create a clear discovery pipeline for investigators. The NIH and CDMRP still coordinate their funding through regular communication so as to avoid overlap. This type of inter‐agency awareness has been critical for NF research, since the total federal spending—although these two agencies provide the highest spending overall for NF1‐MEK‐associated research—is relatively modest relative to other cancers and conditions. For example, between 2008 and 2017, NIH total NF1‐MEK spending was roughly US$51 million, whereas it spent US$541 million for breast cancer research in 2017 alone.^2^ The thoughtful coordination between the NIH and NFRP programs, and appropriate use by researchers of the variety of funding mechanisms, may well have maximized the impact of federal spending, yielding the success that is now being realized for patients.

## The critical role of philanthropic supported research

Since the early 1980s, CTF has focused its relatively limited resources on critical areas for scientific discovery and on expanding the research field to “seed” new ideas and new investigators. To do this effectively, they have worked closely with federal funding agencies to understand trends in funding initiatives for NF, engaged the patient community to ensure that their priorities are being represented in funding strategies, and educated scientists about federal funding and supported research that is critical to NF research but outside of the purview of traditional grant programs. This approach has led to successful follow‐on funding as mentioned above, as well as the growth and integration of an entire research field.

Specifically, the CTF hosts an annual international conference where all funders and stakeholders interact and support smaller awards to generate the data needed to compete for large federal awards. It is reasonable to hypothesize that the steady flow of small awards from CTF fueled the intermittent larger funding commitments by NIH and CDMRP (Fig 2) and that CTF's engagement with all stakeholders helped to shape funding strategies across multiple agencies.

Most recently, NTAP was founded with the mission of accelerating the most promising therapies for pNF and cNF. The narrow focus of NTAP allowed successful partnership with CTF to strategically fund specialized initiatives to move a therapy to the next stage. Similarly, NTAP was able to support critical stages of the clinical development of MEKi for pNF, thereby complementing the efforts of the NIH and CDMRP. The more nimble and specialized focus of these disease‐specific philanthropic funders has maintained and accelerated research at critical moments and provided specialized support for larger initiatives sponsored by federal agencies.

## Intentional integration is feasible and beneficial

CTF and NTAP have been proactively interacting and planning funding strategies together since NTAP's launch. Similarly, NTAP and CTF play active roles in the CDMRP NFRP advisory program and work closely with both the NINDS and NCI NF program leaders. For example, during enrollment for the selumetinib study, NTAP supported patient travel and the opening of two additional study sites while CTF has used its NF patient registry to help identify and recruit participants for research studies. These are some of the tangible examples of the beneficial outcomes of inter‐funder collaboration.

In 2015, the collaborative efforts by the funders were formally expanded via co‐listing NF specific funding in the Dimension for Funders platform. This provides transparency and allows stakeholders to coordinate their efforts, which is particularly valuable for research on rare diseases to maximize resources and to realize clinical benefit. Such integrated funding data also allow funders to identify gaps and overlaps in their investment and foster a community of researchers that has the tools to advance knowledge into clinical research.

Most recently, the NF funding groups have embarked on a new initiative: the largest data sharing effort ever initiated for NF. Spearheaded by CTF in 2014, all NF funders are now incentivizing their applicants—either as a requirement of continued funding, or with the promise of additional funding support—to share their data openly on the recently launched NF data portal (http://www.nfdataportal.org). It is part of the NF Open Science Initiative (NF‐OSI) to support the needs of computational biologists, bench scientists, clinicians, and the public. Participants in the NF‐OSI benefit from early access to results, logistical support for data sharing, and support from data specialists in novel analysis approaches. Thus far, such intentional collaboration across funders has resulted in the launch of new grant mechanisms, integrated analysis of data across initiatives, and the termination of some programs deemed to be duplicative.

## Limitations

There are also limitations to the conclusions that can be drawn from the analysis. NF1 is one rare disease, and the NF1‐MEK funding schemes involves four major funding agencies with clearly stated missions and mandates. Moreover, the leadership of these four funders has been generally constant over a decade which helped to form and maintain such collaborations. Despite these unique circumstances, we feel that the success of this collaborative effort is worthy of consideration by all rare disease communities. The goals of this multi‐part collaboration were to maximize the impact of limited resources; keep patients and families engaged in research; ensure that the research questions being asked are pertinent to patients and likely to be impactful to the field regardless of the study outcome; and prevent silos of data and therefore duplicated efforts, lost time, and wasted resources.

Regardless of the area of study, all research agencies have both strengths and limitations in terms of total funding, flexibility, research scope, speed of action, and reliability of funding from year to year. The collaborations described here balance the relative strengths and limitations of each funder for the maximal benefit of research efficacy and therefore for patients. Such a model of collaboration requires that the leadership of each funding agency is well aware of what their scope and mission is, that they are willing to invest the time to connect and share funding data, and that they are fundamentally open to collaboration. If funders can make that commitment, they payoff is a more integrated and more effective research community.

## Conclusions

As areas of research that require scientific collaboration continue to grow while financial resources remain ever limited across biomedical research, an increase in intentional collaboration across funders has the best prospect of supporting both the initiation and completion of research questions that are most relevant for patients. It also has the highest likelihood of ensuring that precious resources are used responsibly and efficiently. Many times, individual research groups have made great discoveries but lacked the support and incentive to carry on and translate their finding into clinical research. Generally, limited resources and the lack of continuous expertise to progress results from basic research into translational studies, effective clinical design, and trials constitute a major roadblock to the drug development process. That is why by strategizing together, funders can actively identify gaps in the drug development continuum and activate projects in those areas where expertise and resources are missing. What has worked so far for neurofibromatosis can be replicated for other rare diseases if funders are able to take an active, coordinated approach to identify gaps, tools, and infrastructures most needed.

## Conflict of interest

The authors declare that they have no conflict of interest. Opinions, interpretations, conclusions, and recommendations are those of the author/s and are not necessarily endorsed by the Department of Defense or by the National Institute of Health.

## Supporting information

Appendix

LaRosa_grants_data

## Notes

1 Search executed on October 30, 2018.

2 https://www.cancer.gov/about‐nci/budget/fact‐book/data/research‐funding.
