# Supplementary material for: Funding community collaboration to develop effective therapies for neurofibromatosis type 1 tumors
Source: EMBO Mol Med. 2019 Dec 2;12(1):e11656. doi: 10.15252/emmm.201911656 (PMC6949486; doi:10.15252/emmm.201911656)
Supplement: Supplementary file 1 — Appendix [file EMMM-12-e11656-s001.pdf]

Supplementary Information for:

Funding Community Collaboration to Develop Effective Therapies for Neurofibromatosis Type 1 Tumors

Salvatore La Rosa*[1], Vidya Browder[1], Annette C. Bakker[1], Jaishri O. Blakeley[2], Sharad K. Verma[2], Ling M. Wong[3], Jill Morris[3], Naba Bora[4]

[1]Children's Tumor Foundation, New York, NY, USA,

[2]Johns Hopkins University, Baltimore, MD, USA,

[3]National Institute of Neurological Disorders and Stroke, Bethesda, MD, USA,

[4]Congressionally Directed Medical Research Programs, Fort Detrick, MD, USA.

Contents

Methods 2

Broad Research Area (BRA) classification 2

Results analysis 2

  National Institutes of Health. 2

  Neurofibromatosis Research Program (NFRP) 3

  Children's Tumor Foundation 3

  Neurofibromatosis Therapeutic Acceleration Program 4

Methods

To extract all available NF1-MEK funding data, Dimensions for Funders (https://www.dimensions.ai/), a database of publicly and privately funded research projects worldwide, was used. The database was queried using an intersect of two queries that were each specific for MEK inhibitor-related and NF1-related grants. The search was restricted to 2006-2017 to ensure completeness of data among the relevant funders and awards were recorded based on the date the award was made. Results from this search were supplemented using iSearch, a portfolio analysis tool internal to NIH, and Research, Condition, and Disease Categorization (RCDC), a classification scheme used by the NIH for reporting (NIH, 1998) (NIH, 2011). The RCDC classification scheme, which includes intramural and extramural scientific spending across all NIH Institutes and Centers has had a neurofibromatosis category that includes NF1, NF2, and schwannomatosis since 2008. As a result, there was no neurofibromatosis category data from 2006-2008 available for this analysis. iSearch was used to query the neurofibromatosis RCDC category. The integrated output across datasets was manually inspected for relevance to MEK inhibitor development to treat *NF1*, yielding a final curated list of NF1-MEK grants.

Broad Research Area (BRA) classification

The Broad Research Area (BRA) classification implemented in Dimensions was used to distribute the grants into two categories: 'basic research' and 'clinical research'. **Basic Research**: This classification includes research grants from pure basic science to applied research including *in-vitro* studies. **Clinical Research**: This classification includes research grants from *in-vivo* exploratory studies through clinical trials. Several analysis details are notable: 1) Almost all grants are multi-year funding, and the total amount reported here includes the total committed costs as reported at the end of 2018; 2) Foreign grants were converted to US$ based on the exchange rate at the time of the award; 3) Multi-year projects that began before 2006 were not included, even if they were active in 2006 (TABLE 2 in main article).

Results analysis

National Institutes of Health.

The NIH was the leading federal funder of NF1-MEK research, with $51.8 million granted through 29 grants. As NIH does not pre-allocate a specific dollar amount for neurofibromatosis research, all NIH grants included in this analysis had at least one aim that is relevant to NF1 and the development of MEK inhibitors. NIH grants included eleven R01 grants totaling $18.6 million, four National Cancer Institute (NCI) intramural research projects involving clinical trials or natural history studies totaling $18.4 million, a bench-to-bedside award, a Javits award, and several training grants. Funding was approximately evenly distributed between basic and clinical research ($25.1 vs $26.7 million, respectively). Relative to the other NIH institutes and centers, the NCI and the National Institute of Neurological Disorders and Stroke (NINDS) have been

most involved in the development of MEK inhibitors in general and as applied to NF1. Specifically, in 2006-2017 the NCI awarded $33.9 million between its intramural and extramural programs, the NINDS awarded $15.4 million, and remaining NIH funding was provided by the National Institute on Deafness and Other Communication Disorders (NIDCD), National Institute of Arthritis and Musculoskeletal and Skin Diseases (NIAMS), and the National Institute of Dental & Craniofacial Research (NIDCR). In addition to funding several basic research studies involving *NF1*, the NCI has conducted or funded many of the MEK clinical trials, including the first clinical trial to evaluate a MEK inhibitor (CI-1040; NCT00033384 and NCT00034827), the first selumetinib trial (NCT01089101), five other selumetinib trials (NCT01362803, NCT02407405, NCT02839720, NCT03109301, NCT03213691), two other interventional trials (NCT03190915, NCT02465060), and an ongoing natural history study. The NINDS has primarily supported preclinical studies and a national, interdisciplinary center for NF research. NINDS-funded pre-clinical contributions include establishment of a murine model of NF1-related MPNSTs, development of preclinical screening tests to compare potential therapeutics in *NF1* cell lines, and completion of proof-of-efficacy studies for PD0325901. Across all funders, NIH has invested the largest amount across a diversity of research areas related to NF1 and MEK.

Neurofibromatosis Research Program (NFRP)

The NFRP at CDMRP was identified as the second largest federal contributor to NF1-MEK funding, having awarded approximately $35.4 million across 17 grants in 2006-2017 (calendar year of award). Nearly 89% of the dollar amount was directed towards clinical studies. Unlike the NIH, the NFRP allocates a congressionally appropriated budget each year for all forms of NF research. Through the NF Clinical Trials Consortium (NFCTC), the CDMRP has invested more than $27 million in developing all the infrastructure of the consortium and conducting 10 interventional trials, including two trials with MEK inhibitors in pNFs (PD0325901, NCT02096471) and Low-Grade Gliomas (binimetinib, NCT02285439). In addition to initiating clinical trials, the NFRP also funded eight Investigator-Initiated Research Awards ($7.2 million) and five Exploration – Hypothesis Development Awards ($726,504) pertinent to NF1. Other funding mechanisms include the New Investigator Award to encourage non-NF investigators to conduct research in this area, and the Clinical Trial Award (1 award, $540,104). NFRP/CDMRP has also invested in both basic and clinical science, but a there is a notable and unique commitment to funding clinical trials for NF.

Children’s Tumor Foundation

The CTF was the leading non-federal philanthropic funder of NF1-MEK related research 2006-2017, during which time it awarded almost $6.2 million through 34 grants. As the oldest US-based philanthropic foundation dedicated to all forms of NF, CTF has historically acted as a

discovery research seeder and gap funder through its funding mechanisms such as the Young Investigator Award, Drug Discovery Award, and Clinical Research Award. Of the 34 grants, 17 ($1.4 million) were Young Investigator Awards (YIA), ten ($371,123) were Drug Discovery Initiatives (DDI), five ($664,000) were Clinical Research Awards (CRA) and one ($159,500) was a Contract Award (CA). The CTF's Neurofibromatosis Preclinical Consortium (NFPC, funded from 2008-2013) awarded roughly $3.4 million for preclinical studies, followed by the Neurofibromatosis Therapeutic Consortium (NFTC, funded conjointly with NTAP from 2013-2016) that continued funding for $3.6 million. During the 2008-2013 period, the two consortia conducted more than 95 preclinical studies of 38 drugs or drug combinations through collaborations with 18 pharmaceutical companies (Maertens, et al. 2017). Over 40 studies included a MEK inhibitor either as a single agent or in combination. These programs generated critical data in support of MEK as a valid target for *NF1*-driven tumors, providing the preclinical basis that justified the initiation of clinical trials (NCT02096471, NCT02124772, NCT02285439, NCT01362803, NCT02407405) with MEK inhibitors that were then sponsored by either the NFRP/CDMRP or the NIH (Chang, et al. 2013) (Jessen, et al. 2013).

Neurofibromatosis Therapeutic Acceleration Program

although founded only in 2012, has emerged as the second largest non-federal non-profit funder of NF1-MEK research. Focused specifically on accelerating the development of therapeutics for pNF and cNF, since its inception NTAP has teamed up with CTF for the co-sponsoring of preclinical testing through the NFTC consortium. It has also invested over $3.7 million in MEK research, playing a critical role in facilitating the clinical evaluation of selumetinib in children with pNF (NCT02407405).

References

Chang T, Krisman K, Theobald EH, Xu J, Akutagawa J, Lauchle JO, Kogan S, Braun BS, Shannon K (2013) Sustained MEK inhibition abrogates myeloproliferative disease in Nf1 mutant mice. J Clin Invest 123: 335 – 339

Jessen WJ, Miller SJ, Jousma E, Wu J, Rizvi TA, Brundage ME, Eaves D, Widemann B, Kim MO, Dombi E et al (2013) MEK inhibition exhibits efficacy in human and mouse neurofibromatosis tumors. J Clin Invest 123: 340 – 347 6 5

Maertens O, McCurrach ME, Braun BS, De Raedt T, Epstein I, Huang TQ, Lauchle JO, Lee H, Wu J, Cripe TP et al (2017) A collaborative model for accelerating the discovery and translation of cancer therapies. Can Res 77: 5706 – 5711
